# A pilot study on protocol consistency and graph metric reproducibility in microstructure-weighted connectomes

**DOI:** 10.1038/s41598-026-38964-z

**Published:** 2026-02-11

**Authors:** Maddalena Cavallo, Mattia Ricchi, Aaron Axford, Kylie Yeung, Jordan McGing, Leonardo Brizi, Damian J. Tyler, Claudia Testa, James T. Grist

**Affiliations:** 1 Department of Physics and Astronomy, University of Bologna, Bologna, Italy; 2 Oxford Centre for Clinical Magnetic Resonance Research, University of Oxford, Oxford, UK; 3 Department of Computer Science, University of Pisa, Pisa, Italy; 4 Division of Bologna, National Institute of Nuclear Physics (INFN), Bologna, Italy; 5 Department of Oncology, University of Oxford, Oxford, UK; 6 Department of Radiology, Oxford University Hospitals NHS Trust, Oxford, UK; 7 Department of Physiology, Anatomy, and Genetics, University of Oxford, Oxford, UK

**Keywords:** Brain structural connectivity, Weighted connectomes, Reproducibility, Diffusion MRI, Bingham-NODDI, Graph metrics, Biomarkers, Computational biology and bioinformatics, Neurology, Neuroscience

## Abstract

Microstructure-weighted connectomes incorporate diffusion parameters into structural networks, offering a rich characterisation of brain connectivity. While these biologically-informed connectomes have shown sensitivity to pathology-related alterations (for example in multiple sclerosis), their reproducibility remains largely unexplored. In this study, we evaluated the consistency of connectomes weighted with tensor and Bingham-NODDI parameters, employing a four-shell acquisition protocol to ensure accurate fibre reconstruction. Phantom and *in vivo* (N=4) data were acquired to assess temporal, inter-site and inter-protocol reproducibility of weighting parameters and inter-site stability of graph metrics. High reproducibility was observed for fractional anisotropy (FA), mean diffusivity (MD), and intra-neurite (INVF) and intra-cellular (ICVF) volume fractions, with coefficients of variation (CVs) below 5% and negligible Bland-Altman biases. Orientation dispersion index and $\beta$ concentration parameter showed CVs above 5% and were excluded from connectome construction. Graph metrics extracted from FA-, MD- and INVF-weighted connectomes exhibited good consistency, except for modularity. Extra-cellular volume fraction (ECVF)-weighted connectomes showed poor reproducibility (CV>5%, intraclass correlation coefficient <0.5). These preliminary findings demonstrate the reliability of microstructure-weighted connectomes, identifying the weighting strategies and graph metrics with the highest reproducibility. This supports the use of network metrics derived from weighted connectomes as potential biomarkers of altered brain connectivity in neurological disorders.

## Introduction

Structural connectivity in the human brain can be non-invasively reconstructed using diffusion Magnetic Resonance Imaging (dMRI) and tractography algorithms^1^. In white matter (WM), the coherent organisation of myelinated axons into fibre bundles induces anisotropic diffusion of water molecules, which can be characterised *in vivo* by acquiring diffusion-weighted signals^2,3^. Tractography algorithms integrate this local orientation information to infer the long-range streamlines representing WM fibre pathways^4^. These reconstructed pathways can be quantitatively analysed either by focusing on specific anatomical bundles (tract-specific analysis) or by considering the entire brain tractogram (connectome-based analysis)^1^. In particular, the connectome-based approach characterises patterns of whole-brain connectivity by constructing the connectome - a matrix describing the structural connections between pairs of grey matter (GM) regions^1^. The connectome can be modelled as an undirected network^5^, with nodes representing GM regions and edges representing their connections in terms of WM fibres. Graph theory offers a powerful framework for analysing the properties of this network, characterising its complexity with simple descriptors^6^. Structural connectivity alterations have been observed in healthy ageing^7,8^ as well as in different neurological conditions, including multiple sclerosis^9^, Alzheimer’s disease^10^ and mild cognitive impairment^11^. Furthermore, structural connectivity has been shown to closely relate to functional connectivity^12^.

Connectome edges are commonly weighted with the number of streamlines (NOS). However, this method lacks biological specificity, as the number of streamlines does not directly reflect the underlying fibre density, but is rather a reconstruction of tractography algorithms^3,13,14^. Post-processing approaches for diffusion data such as Diffusion Tensor Imaging (DTI)^15^ and Neurite Orientation Dispersion and Density Imaging (NODDI)^16^ provide various voxel-wise parameters that describe microstructural properties and the integrity of both fibres and surrounding tissues^17^. Therefore, integrating these parameters into structural connectomes allows for the construction of biologically enriched networks^13^. These microstructure-weighted connectomes have shown significant associations with resting-state functional connectivity in healthy subjects^18^ and have demonstrated high sensitivity to alterations caused by neurological diseases. In particular, network metrics derived from DTI-, NODDI- and g-ratio-weighted connectomes have been shown to characterise multiple sclerosis and associate with disease progression and clinical disability^19–23^. Furthermore, connectomes weighted with Diffusion Kurtosis Imaging (DKI) and NODDI parameters have shown the ability to discriminate Parkinson’s disease patients from healthy controls^24^, and NODDI-weighted connectomes have demonstrated sensitivity to epilepsy^25^. Finally, structural connectomes weighted with fractional anisotropy (FA) have been shown to characterise Alzheimer’s disease^26^ and schizophrenia^27^.

The reproducibility of graph metrics should be assessed before their use as clinical biomarkers^28^. The topological properties of structural connectomes are highly sensitive to the methodological choices made throughout the reconstruction pipeline, including preprocessing steps, acquisition schemes, tractography algorithms and brain parcellation strategies^1,13,28,29^. Moreover, variability across imaging sites and protocols can further affect the consistency of connectome measures^5,28^. Therefore, it is crucial to evaluate the robustness of the connectome when selecting a specific reconstruction strategy^13^. Microstructure-weighted connectomes introduce an additional layer of complexity, as their robustness also depends on the consistency of the diffusion model parameters used to define edge weights. While several studies have investigated the robustness of NOS-based connectomes (see Welton et al.^28^ for a review), to our knowledge, no study has systematically assessed the reproducibility of microstructure-weighted connectomes that incorporate DTI and NODDI parameters. Specifically, the reproducibility of NOS-weighted connectomes has been examined across different diffusion encoding strategies^30^, imaging sequences^31^ and tractography algorithms^32,33^. Nevertheless, the reproducibility of these connectomes is potentially misleading, as NOS lacks direct biological interpretation and is inherently dependent on user-defined tractography parameters^3,13,14^. A few studies have also addressed the reproducibility of FA-weighted connectomes derived from single-shell^34^, two-shell^35^ and three-shell^36^ datasets. However, the reproducibility of more advanced microstructural weightings, such as those derived from multi-compartment models like NODDI, remains largely unexplored.

In this context, this work presents the first comprehensive validation of microstructure-weighted connectomes constructed using tensor and Bingham-NODDI^37^ model parameters, integrating phantom and *in vivo* data to assess temporal, inter-site, and inter-protocol reproducibility. While two-shell acquisition schemes are sufficient for fitting the NODDI model^37^, more than two shells are required to accurately reconstruct the structural connectome^38^. In this study, a four-shell HARDI (High Angular Resolution Diffusion Imaging) acquisition scheme was employed to achieve a balance between accurate fibre reconstruction and acquisition time. First, diffusion model parameters extracted from four-shell protocol data were evaluated for temporal and inter-site consistency using phantom and *in vivo* acquisitions. This aimed to identify the most reliable parameters for weighted connectome reconstruction and to minimise variations in connectome metrics caused by unstable weighting. This step is particularly important for Bingham-NODDI parameters, which have shown lower reliability compared to DTI metrics^39^, possibly due to the greater complexity of the model. Furthermore, results from four-shell protocol were compared with those obtained using the two-shell protocol commonly used for fitting the NODDI model. Connectomes were then reconstructed from four-shell protocol data and weighted using diffusion model parameters. The robustness of these microstructure-weighted connectomes was assessed by evaluating the stability of the derived network metrics across acquisitions performed at different sites. The validation of these connectomes is a crucial step toward their reliable use for investigating brain properties in both healthy and pathological conditions.

## Methods

### Acquisition protocols

Diffusion-weighted images were acquired using a single-shot, spin-echo diffusion-weighted sequence, with echo planar imaging (EPI) readout. The sequence included a single $180^{\circ }$ refocusing pulse and a repetition time (TR) of 5000 ms, allowing for the acquisition of multiple slices within a single TR. The field of view (FOV) was 240 mm$\vphantom{0}^2$, and acquisition matrix 96$\times$96, with slice thickness 2.5 mm, simultaneous multi-slice factor 2, and ASSET (Array Spatial Sensitivity Encoding Technique) factor 2. Multi-shell diffusion acquisition protocols were used in this study. Specifically, a two-shell protocol and a four-shell protocol were employed, as detailed in the following subsections. All acquisitions were performed using two 3T scanners (SIGNA Premier, General Electric Healthcare, WI, USA) equipped with 21-channel head and neck coils.

#### Two-shell diffusion protocol

The two-shell protocol is a multi-shell protocol that includes two distinct b-values and 99 gradient directions. In particular, diffusion weighted signals were acquired with a b-value of 1000 s/mm$\vphantom{0}^{2}$ along 30 directions and a b-value of 2600 s/mm$\vphantom{0}^{2}$ along 60 directions. The echo time (TE) was 70.1 ms. Additionally, nine b = 0 s/mm$\vphantom{0}^{2}$ images were randomly dispersed between the diffusion-weighted images. Diffusion-weighted images were acquired with the phase encoding gradient in the anterior-posterior direction. Total acquisition time for the two-shell protocol was 8 minutes and 25 seconds.

#### Four-shell diffusion protocol

The four-shell protocol included b-values of 700, 1000, 2000, 3000 s/mm$\vphantom{0}^{2}$^22^. B-values were chosen to be lower than 3500 s/mm$\vphantom{0}^{2}$, as this range has been demonstrated to optimise the balance between fibre angular resolution and signal-to-noise ratio^14,38^. A total of 150 volumes were acquired, distributed as follows: 6 gradient directions for b = 700 s/mm$\vphantom{0}^{2}$, 20 directions for b = 1000 s/mm$\vphantom{0}^{2}$, 45 directions for b = 2000 s/mm$\vphantom{0}^{2}$ and 66 directions for b = 3000 s/mm$\vphantom{0}^{2}$. Thirteen directions of the gradient were acquired with no diffusion weighting (b = 0 s/mm$\vphantom{0}^{2}$). The TE was 72.4 ms. Total acquisition time for the four-shell protocol was 12 minutes and 40 seconds.

#### Structural acquisition protocol

A T1-weighted acquisition was included in the imaging protocol for the *in vivo* scans. Whole brain T1-weighted volumes were acquired using a 3D Magnetization Prepared RApid Gradient Echo (MP-RAGE) sequence. The acquisition parameters were set as follows: TR = 6 ms, Inversion Time = 950 ms, TE = 3.2 ms and Flip Angle = $8^{\circ }$. The FOV was 300 mm$\vphantom{0}^2$, with a slice thickness of 1 mm. T1-weighted images were acquired for each subject in the same session as the diffusion volumes.

### Phantom and healthy volunteers

A phantom was used in this study, as it provides controlled and well-defined environment for fitting diffusion models and ensuring the reliability of tractography reconstructions. A phantom with similar composition and diffusion properties has already been validated as a reliable proxy for human brain tissues^40^. The phantom consists of two fibre strands, made from polyfill fibres (50 dtex, composed of multiple 15$\mu$m fibres, Filamentgarn TYPE 611, Trevira GmbH, Bobingen, Germany), which are wound around a spherical spindle^41^. Water is present between the fibres, simulating the restricted anisotropic diffusion characteristics of WM. The fibres cross each other at $60^{\circ }$ angle, and have a reported^41^ FA of $0.78 \pm 0.02$. This crossing fibre configuration effectively mimics the behaviour of WM bundles observed in the human brain. The spherical spindle is immersed in a fluid made of a mixture of distilled water and sodium chloride (83 g NaCl per kilogram of water). The concentration of NaCl is adapted to minimise the susceptibility difference between the fluid and fibres and to eliminate orientation-dependent transversal relaxation times^41^. The phantom was scanned three times on the same day with each multi-shell protocol to study the stability of fit results over time.

Healthy volunteers were recruited for this study. All experiments were performed in accordance with the relevant guidelines and regulations. Written informed consent was obtained from all subjects prior to their inclusion in the study. Data were collected under the ethically approved protocol “LEARN: VoLunteEr scanning to develop and optimise mAgnetic ResonaNce imaging” (IRAS reference: 302326). Four volunteers were scanned (three females, one male, mean age±std: 27±4 years), all reporting no prior history of neurological or psychiatric conditions. Each participant was scanned at two hospitals within a 10-day interval to assess inter-centre reproducibility of the results. The same setup (scanner, coils) and acquisition sequences were used in both hospitals, to identify possible systematic differences between the sites while using the same configuration.

### Diffusion parameter estimation

#### Distortion correction

Raw diffusion-weighted images were preprocessed to correct for distortions caused by susceptibility-induced and eddy current-induced off-resonance fields. These corrections were performed using tools from the FMRIB Software Library (FSL)^42^, integrated in Python via the fslpy package. In particular, the topup tool^43^ was employed to estimate the susceptibility-induced off-resonance field and reconstruct the unwarped image by leveraging volumes acquired with opposing phase-encoding polarities. Images without diffusion weighting (i.e. b-value = 0) were used for this step, as they offer a higher signal-to-noise ratio and are not affected by eddy current distortions caused by diffusion gradients. Subsequently, brain-only masks were generated using the Brain Extraction Tool (BET)^44^ from FSL. The eddy tool^45^ was then employed to correct for eddy current-induced distortions and subject motion.

#### Diffusion models implementation

Pre-processed diffusion-weighted images were fit with the tensor model using the FSL function dtifit. This command estimates the three eigenvectors $\epsilon _1, \epsilon _2, \epsilon _3$ and associated eigenvalues $\lambda _1, \lambda _2, \lambda _3$ of the diffusion tensor for each voxel, representing principal directions of diffusion and relative apparent diffusivities, respectively. The estimated eigenvalues are then used to compute the Mean Diffusivity (MD) and Fractional Anisotropy (FA) quantitative maps. The NODDI model was implemented with the DMIPY (Diffusion Microstructure Imaging in Python) software package^46^. NODDI is a two-level multi-compartment framework that models the acquired signal as the sum of the contributions of tissue and non-tissue components. It can be implemented in DMIPY as shown in Eq. (1), where $f_{CSF}$, $f_{extra}$ and $f_{intra}$ represent the volume fractions of non-tissue, extra-neurite and intra-neurite components, respectively. In this work, the isotropic diffusivity $\lambda _{CSF}$ was fixed at $3.0 \times 10^{-9} m^{2}/s \,$, while the neurite intrinsic diffusivity $\lambda _\parallel$ was set to $1.7 \times 10^{-9} m^2/s \,$^37^. The Bingham distribution^47^ was chosen as the orientation distribution function of the neurites, as it can model anisotropic diffusion about the dominant orientation^37^. Quantitative maps of volume fractions, concentration parameter $\beta$ and Orientation Dispersion Index (ODI) were extracted as outputs. Additionally, mean squared error (MSE) maps were extracted to assess fit quality.

1 $$\begin{aligned} Bingham-NODDI = \underbrace{ f_{\text {CSF}} \overbrace{\text {G1}(\cdot \vert \lambda _{\text {CSF}})}^{\text {Ball}}}_{\text {CSF}} + \overbrace{\text {SD2}(\kappa _1, \kappa _2, \mu , \psi )}^{\text {Bingham}} *_{S^2} \left[ \underbrace{ f_{extra} \overbrace{\text {G2}(\cdot \vert \lambda _{\perp }^{\text {tort}}, \lambda _{\parallel })}^{\text {Zeppelin}} }_{\text {Extra-Neurite}} + \underbrace{ f_{intra} \overbrace{\text {C1}(\cdot \vert \lambda _{\parallel })}^{\text {Stick}} }_{\text {Intra-Neurite}} \right] \end{aligned}$$

#### Spatial registration and ROIs definition

**Fig. 1:**
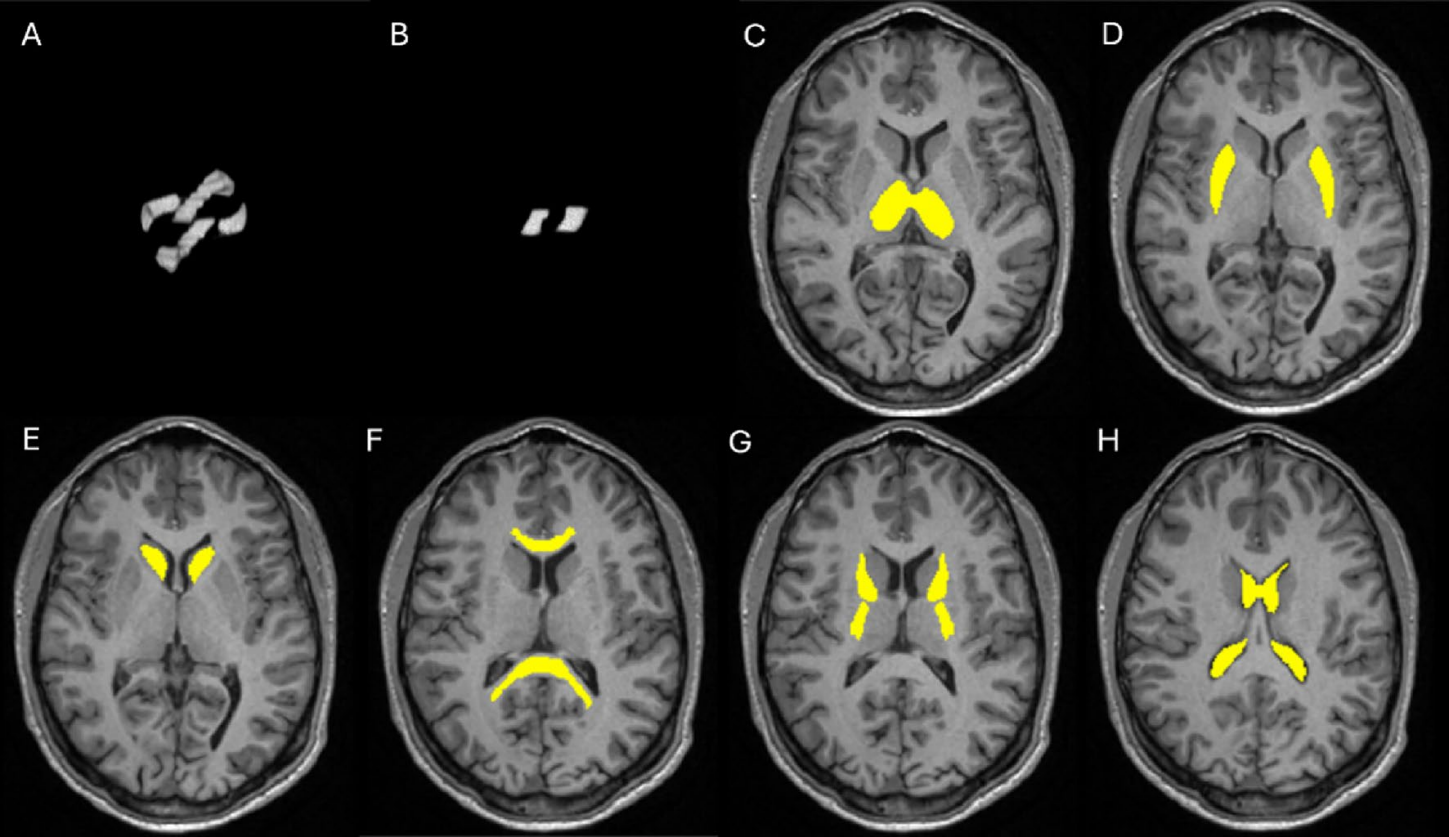
Regions of Interest (ROIs) designed to extract tensor and Bingham-NODDI model parameters. Brain ROIs were generated using atlases available in FSL and applying an erosion to avoid partial volume effects. (**a**) Phantom ring-only regions (**b**) Phantom crossing regions (**c**) Thalamus (**d**) Putamen (**e**) Caudate (**f**) Genu and Splenium of the Corpus Callosum (**g**) Anterior and Posterior Limb of the Internal Capsule (**h**) Ventricles.

Quantitative parameter maps obtained from *in vivo* data were registered to the MNI (Montreal Neurological Institute) standard space. To achieve this, T1-weighted images were first registered to standard space using flirt (FMRIB’s Linear Image Registration Tool)^48^ and fnirt (FMRIB’s Nonlinear Image Registration Tool)^49^ commands from FSL. The resulting warp field was then applied to register the parameter maps to the MNI, using the applywarp command with trilinear interpolation. Different Regions of Interest (ROIs) were designed to extract tensor and Bingham-NODDI parameters from specifically selected regions, as shown in Fig. 1. Two ROIs were defined to extract the results from phantom acquisitions. The first ROI encompasses the ring region only, while the second ROI covers the crossing region of the two fibres. Brain ROIs were generated from the Harvard-Oxford Atlas (RRID:SCR_001476) available in FSL. Six different ROIs were defined, covering the following regions: thalamus, putamen, caudate, genu and splenium of the corpus callosum, anterior and posterior limbs of the internal capsule, and ventricles. Brain ROIs were eroded using the fslmaths -ero command (kernel size $3\times 3\times 3$) in order to minimise partial volume effects. Mean values and standard deviations of the parameters were extracted in each ROI defined.

### Structural connectome reconstruction

Structural connectomes were reconstructed from *in vivo* data acquired with the four-shell protocol. Connectome construction procedure involves several steps, implemented with MRtrix3^50^. Initially, PCA-based denoising^51^ was performed on the raw diffusion-weighted volumes. Distortion correction was then applied to the denoised images using topup and eddy tools, following the procedure explained in the previous section. Fibre orientation density functions were estimated in each voxel through a multi-shell multi-tissue constrained spherical deconvolution (MSMT-CSD)^52^ of the acquired signal. Probabilistic tractography was performed using the iFOD2 (second-order integration over fibre orientation distributions) algorithm^53^. To improve biological plausibility, anatomically constrained tractography (ACT)^54^ was employed, generating a total of 3 million streamlines seeded from the WM/GM interface. The resulting streamlines were weighted using the SIFT2 (spherical-deconvolution informed filtering of tractograms)^55^ algorithm to improve the quantitative accuracy of the reconstruction. Structural connectomes were then constructed from the generated tractogram and modelled as graphs. Network nodes were defined according to FreeSurfer^56^ parcellations of T1-weighted images, based on the Desikan-Killiany^57^ cortical atlas. Specifically, the recon-all command was employed, resulting in eighty-four distinct brain regions. Network edges were defined by assigning streamlines to these parcels using the tck2connectome^58^ command. Edges were weighted with diffusion model parameters, namely FA and MD from the tensor model and intra-neurite volume fraction (INVF) and extra-cellular volume fraction (ECVF) from the NODDI model. The ODI and $\beta$-fraction were excluded, as the reproducibility analysis showed high instability for these parameters. Consequently, four distinct weighted connectivity matrices were obtained for each subject. A tractometry-based^59^ approach was adopted to compute edge weights for each connectome. The diffusion parameter map was sampled at each streamline vertex using tcksample to determine the median value per streamline. As suggested by Boshkovski et al.^60^, the median was preferred over the mean as it is more robust to outliers and makes no assumptions about the distribution of values along the bundle. Subsequently, the final weight for each edge was computed as the weighted mean of these per-streamline median values across all streamlines assigned to that edge, using the corresponding SIFT2 coefficients as weights. This ensures that streamlines representing greater fibre density (i.e., those with large SIFT2 weights) contribute proportionally more to the edge weight calculation, resulting in a more biologically accurate connectome estimation. Since the edge weights integrated SIFT2 contributions, no further thresholding or pruning was applied to the weighted connectomes^13,61^, preserving the full topological information of the network. Connectome matrices were made symmetrical, as the direction of diffusion cannot be resolved with dMRI techniques^5^. Various metrics were extracted from the weighted connectomes to study network properties, including measures of integration (density, global efficiency, nodal strength) and segregation (clustering coefficient and modularity). These network metrics were extracted using the Brain Connectivity Toolbox^6^, employing weighted versions when available. Clustering coefficient and nodal strength were averaged across all nodes to provide a single output value. Further processing was required for MD- and ECVF-weighted connectomes. Network analysis requires that edges with the highest weights represent the strongest connections^62^ but this assumption fails in MD- and ECVF-weighted connectomes. To avoid this issue, the negative logarithmic transformation was applied to each entry of these weighted connectomes before metrics extraction^1,22,62^.

### Statistical analysis

Statistical analysis was performed to assess the reproducibility of results across acquisitions performed under different conditions (repeated scans, different acquisition sites, different protocols). Specifically, the coefficient of variation (CV), Bland-Altman (BA) analysis and intraclass correlation coefficient (ICC) were computed. The CV was employed to evaluate intra-subject variability. It measures the relative dispersion of a dataset and is computed as the ratio of its standard deviation to the mean. The CV is typically reported as a percentage, and a threshold of 5% was chosen in this study to indicate low relative variability and distinguish acceptable from non-acceptable fluctuations. Furthermore, Bland-Altman analysis^63^ was performed to identify systematic biases across the results. This method is widely used to assess the agreement between two different measurements of the same medical parameter^64–67^. The Bland-Altman bias represents the average of the differences between paired measures and is computed as shown in Eq. (2), where $X_{1,i}$ and $X_{2,i}$ denote the *i*-th measurement obtained from the first and second method, respectively. The limits of agreement (LoA), providing the 95% confidence interval (CI), are computed as shown in Eq. (3). A Bland-Altman bias close to zero, along with narrow limits of agreement, indicates the absence of systematic differences between measurements.

2 $$\begin{aligned} \text {BA bias} = \frac{1}{n} \sum _{i=1}^{n} (X_{1,i} - X_{2,i}) \end{aligned}$$

3 $$\begin{aligned} \text {LoA} = \text {BA bias} \pm 1.96 \sigma \end{aligned}$$

Finally, the ICC^68^ was computed to quantify the reliability of the measurements. The ICC estimates the proportion of the total observed variance that is attributable to true differences between subjects. Following the guidelines by Koo et al.^69^, in this study we selected the two-way random-effects model, with single measure and absolute agreement. This specific formulation of the ICC is denoted as ICC(2,1) and is calculated as reported in Eq. 4, where MS$\vphantom{0}_S$ represents the mean square between subjects, MS$\vphantom{0}_E$ the mean square for error, MS$\vphantom{0}_R$ the mean square between raters, *n* the number of subjects, and *k* the number of raters. In this study, the raters correspond to the acquisition sites. The ICC values range from 0 (no agreement) to 1 (perfect agreement). Reliability is commonly interpreted as follows: values less than 0.5 indicate poor reliability, between 0.5 and 0.75 moderate, between 0.75 and 0.9 good, and greater than 0.9 excellent reliability^69^. The ICC was computed using the Pingouin^70^ package in Python.

4 $$\begin{aligned} ICC(2,1) = \frac{\textrm{MS}_S - \textrm{MS}_E}{\textrm{MS}_S + (k-1)\textrm{MS}_E + \frac{k}{n}\left( \textrm{MS}_R - \textrm{MS}_E\right) } \end{aligned}$$

## Results

### Four-shell protocol stability

#### Temporal stability

Temporal stability of DTI and Bingham-NODDI parameters was assessed by performing three consecutive scans on a phantom^41^. FA and MD were extracted from the tensor model fit in the two ROIs defined. The CV was computed to assess if there was a consistent difference among the scans performed with the four-shell protocol. CVs were found to be lower than 0.50% in both the ring and the crossing ROIs, with most of the parameters presenting minimal variation among scans (CV = 0% when considering two significant digits). In particular, FA was found to have a consistent value equal to $0.70 \pm 0.08$ in the ring-only ROI and equal to $0.51 \pm 0.05$ in the crossing-only ROI.

**Fig. 2:**
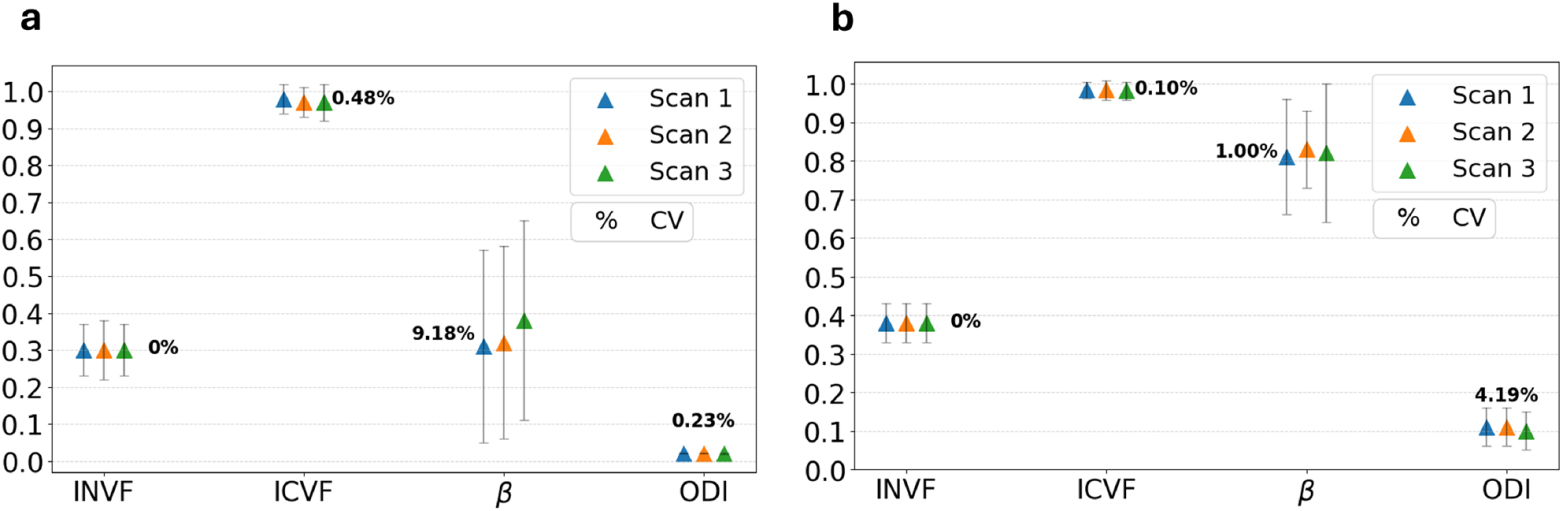
Temporal consistency of Bingham-NODDI parameters extracted from four-shell protocol data. INVF: Intra-Neurite Volume Fraction, ICVF: Intra-Cellular Volume Fraction, $\beta$: concentration parameter, ODI: Orientation Dispersion Index, CV: Coefficient of Variation. CV values equal to 0% for INVF indicate no variation among scans when considering two significant digits. (**a**) Phantom ring ROI (**b**) Phantom crossing ROI.

Quantitative maps of ODI, $\beta$ concentration parameter and volume fractions were extracted from Bingham-NODDI fit. As for the tensor model fit, results were extracted from those maps applying two binary masks, focused on the ring-only and crossing-only regions of the phantom. The resulting mean values of the parameters and associated standard deviations for each scan and ROI are summarised in Fig. 2, together with the computed CVs. The CV among scans was found to be lower than 0.50% for the INVF and ICVF parameters in both ROIs. ODI and $\beta$ concentration parameters presented larger temporal fluctuations. Specifically, the $\beta$ concentration parameter exhibited a CV of 9.18% in the ring-only ROI, while the ODI parameter had a CV of 4.19% in the crossing ROI. The MSE was found to be equal to $0.0007 \pm 0.0004$ in the ring ROI and equal to $0.0017 \pm 0.0005$ in the crossing ROI.

Diffusion models parameters were extracted from two-shell protocol data and compared among scans. The measured values of FA and MD were found to be stable in time in both ROIs, with CVs lower than 0.90%. Bingham-NODDI parameters exhibited good stability in both ROIs, with the exception of the ODI index in the crossing region (CV = 7.07%). Unlike the four-shell protocol, the $\beta$-fraction demonstrated a good inter-scan reproducibility, with CVs of 1.14% and 1.49% in the ring and crossing ROI, respectively.

#### Inter-site reproducibility

**Fig. 3:**
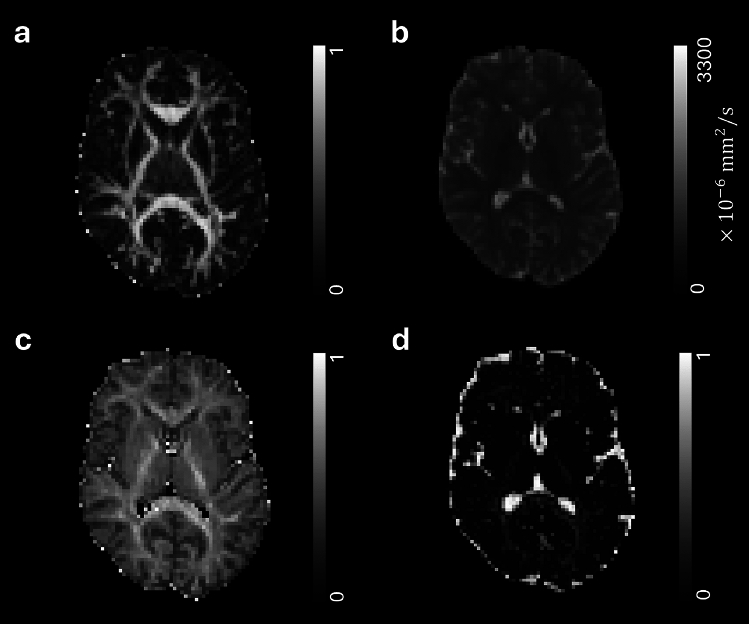
Examples of quantitative parameter maps derived from tensor and Bingham-NODDI fit on *in vivo* data. These maps will be used to weight connectome edges, generating four distinct microstructure-weighted connectomes for each subject. (**a**) Fractional Anisotropy (**b**) Mean Diffusivity (**c**) Intra-Neurite Volume Fraction (**d**) Extra-Cellular Volume Fraction.

**Fig. 4:**
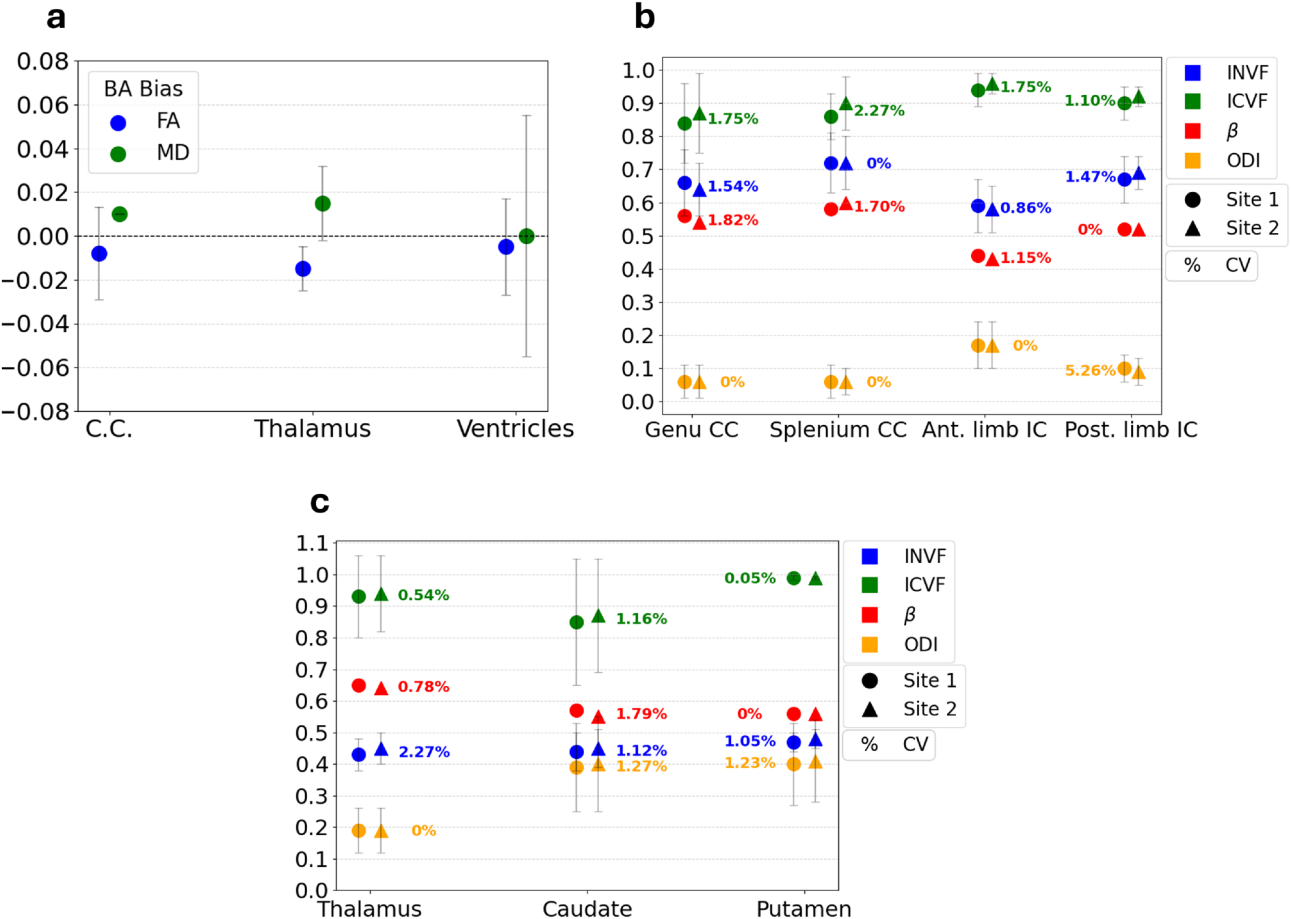
Inter-site reproducibility of diffusion models parameters extracted from four-shell protocol data. (**a**) Bland-Altman bias and 95% confidence interval for FA and MD from tensor model fit, in Corpus Callosum (C.C.), Thalamus and Ventricles. (**b**) Bingham-NODDI parameters and associated coefficients of variation (CV) among sites for one subject in WM ROIs (**c**) Bingham-NODDI parameters and associated CVs among sites for one subject in GM ROIs. $\beta$ parameter error bars are not shown in the plot to improve visualisation. CV values equal to 0% indicate no variation among scans when considering two significant digits.

Inter-site reproducibility of four-shell protocol data was evaluated by scanning healthy volunteers at two sites within 10 days. Both sites used identical 3T GE scanners, 21-channel head and neck coils, and acquisition sequences to minimise variability and isolate systematic differences. Fig. 3 (a-b) presents an example of the quantitative maps of FA and MD obtained from fitting *in vivo* data with tensor model. As can be appreciated in the figure, the highest FA value was found in the corpus callosum, highlighting its highly oriented structure. The lowest FA and the largest MD was found in the ventricles, reflecting their isotropic diffusion environment. Results were extracted from these maps using three ROIs, namely corpus callosum, thalamus and ventricles. Inter-site reproducibility of the obtained values was assessed by computing the CV between the two sites. FA exhibited a CV lower than 5% across all subjects. More specifically, it was found to be less than 1.54% in the corpus callosum, less than 3.45% in the thalamus, and less than 3.70% in the ventricles. Regarding MD, the CV across sites was found to be lower than 2% in every subject (CV$\vphantom{0}_{CC}$ $\le$ 0.81%, CV$\vphantom{0}_{Thal}$ $\le$ 1.61%, CV$\vphantom{0}_{Ventricles}$ $\le$ 1.29%). Bland-Altman analysis was performed on the parameters extracted in each ROI to test the presence of a bias across acquisition sites. Fig. 4 (a) shows the Bland-Altman bias together with the 95% CI for FA and MD in the three ROIs. The observed Bland-Altman bias was on the order of 10$\vphantom{0}^{-2}$ to 10$\vphantom{0}^{-3}$ across all ROIs, with zero included in the majority of the 95% CIs. Lastly, inter-site ICCs demonstrated moderate to excellent reliability^69^ for FA and MD across all investigated ROIs (see Supplementary Table S1).

Fig. 3 (c-d) presents an example of the quantitative parameter maps derived from Bingham-NODDI fit on *in vivo* data. Seven ROIs were designed to extract the results from these maps, in both WM (genu and splenium of the corpus callosum, anterior and posterior limbs of the internal capsule) and GM (thalamus, caudate and putamen). Mean values obtained in each ROI were compared across sites by computing the CV, separately for each subject. Fig. 4 (b-c) shows an example of the extracted results for one subject, in WM and GM ROIs. Comparable results were obtained from the other subjects. As expected, the INVF was found to be lower in GM ROIs compared to WM ROIs, with values typically above 0.5 in the latter. Concerning fit quality, MSE was on the order of 10$\vphantom{0}^{-3}$ in all the ROIs for all *in vivo* data. The analysis revealed CVs among sites smaller than 2.3% for INVF and ICVF in all the ROIs. Additionally, the $\beta$ concentration parameter and ODI generally demonstrated good agreement across sites, with most CVs below the 5% threshold. However, isolated instances of higher variability were observed (e.g., ODI in the posterior limb of the internal capsule in Fig. 4 (b)). Inter-site ICCs are presented in Supplementary Table S1. INVF exhibited moderate to excellent reliability in WM ROIs, while showing lower reliability in GM ROIs. $\beta$ and ODI demonstrated consistently high ICC values across most ROIs, while ICVF exhibited a more heterogeneous profile. The lower ICC values observed in some ROIs for ICVF may be attributable to the limited inter-subject variability of this parameter in healthy subjects.

**Fig. 5:**
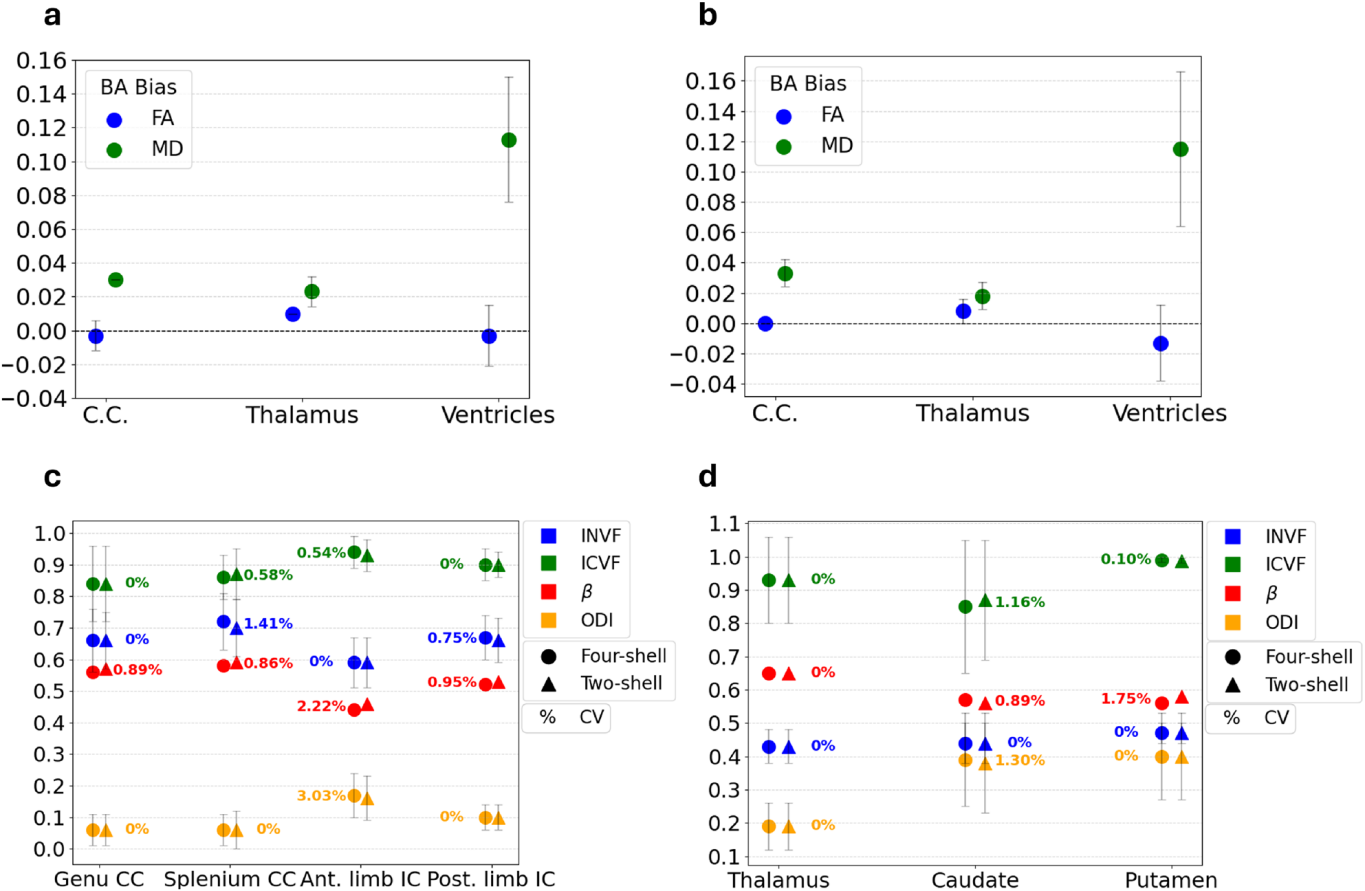
Multi-shell protocol comparison using tensor and Bingham-NODDI parameters. (**a** - **b**) Bland-Altman bias and 95% confidence intervals for DTI parameters extracted in the two acquisition sites. Positive bias indicates larger values obtained with the two-shell protocol. (**c** - **d**) Bingham-NODDI parameters and associated coefficients of variation (CV) between protocols in WM and GM ROIs, respectively. CV values equal to 0% indicate no variation among protocols when considering two significant digits of the parameters.

### Multi-shell protocol comparison

Phantom and *in vivo* data were also acquired using a two-shell protocol. Data were fit with the tensor and Bingham-NODDI models and analysed using the same ROIs previously described. The aim was to evaluate the accordance of the obtained results with those of the four-shell protocol. For the phantom data, mean values across the three consecutive scans were computed in each ROI. These averages were then compared across protocols. For the *in vivo* analysis, data from both acquisition sites were included in the protocol comparison to ensure a larger dataset. Tensor model parameters generally exhibited small variations among the protocols. In the phantom, FA showed a CV equal to 0% in the rings ROI (considering two significant digits) and to 1.29% in the crossing ROI. Similarly, the CV for MD was 1.41% in the ring ROI and 2.10% in the crossing ROI. When considering *in vivo* data, the highest CV values observed across all subjects for FA were 0.80% in the corpus callosum, 1.75% in the thalamus, and 5.66% in the ventricles, while those for MD were 3.08%, 2.16% and 4.97%, respectively. In order to further understand if there was a bias between the tensor parameters extracted with the two protocols, Bland-Altman analysis was performed. The Bland-Altman bias was computed by subtracting four-shell protocol results from two-shell protocol ones. Thus, a positive bias indicates a larger estimate obtained with the two-shell protocol. The obtained BA bias and limits of agreement are shown in Fig. 5 (a-b), separately for each acquisition site. Overall, high consistency was found in tensor model parameters extracted using the two protocols, with the exception of MD in the ventricles.

Subsequently, the Bingham-NODDI parameters extracted from the two protocols were compared. MSE values obtained from the two-shell protocol were on the order of $10^{-3}$, consistent with the results of the four-shell protocol. This indicates that the Bingham-NODDI model generally fits data acquired with the two protocols equally well. In the phantom, INVF and ICVF exhibited low CVs across protocols, with values equal to 1.64% and 0.69% in the ring ROI, and equal to 0.44% and 0.22% in the crossing ROI, respectively. Conversely, $\beta$ and ODI values presented large variations, with peaks of 10.22% in the rings ROI for $\beta$ and 23.08% in the crossing ROI for ODI. Fig. 5 (c, d) shows an example of the inter-protocol CVs obtained for Bingham-NODDI parameters on *in vivo* data. In contrast to the phantom findings, *in vivo* results demonstrated good inter-protocol accordance across all parameters (including $\beta$ and ODI), with CVs remaining below the 5% threshold. Bland-Altman analysis revealed minimal systematic bias and narrow limits of agreement across all parameters and ROIs, as detailed in Supplementary Table S2 and S3.

### Parameter-weighted connectomes

**Fig. 6:**
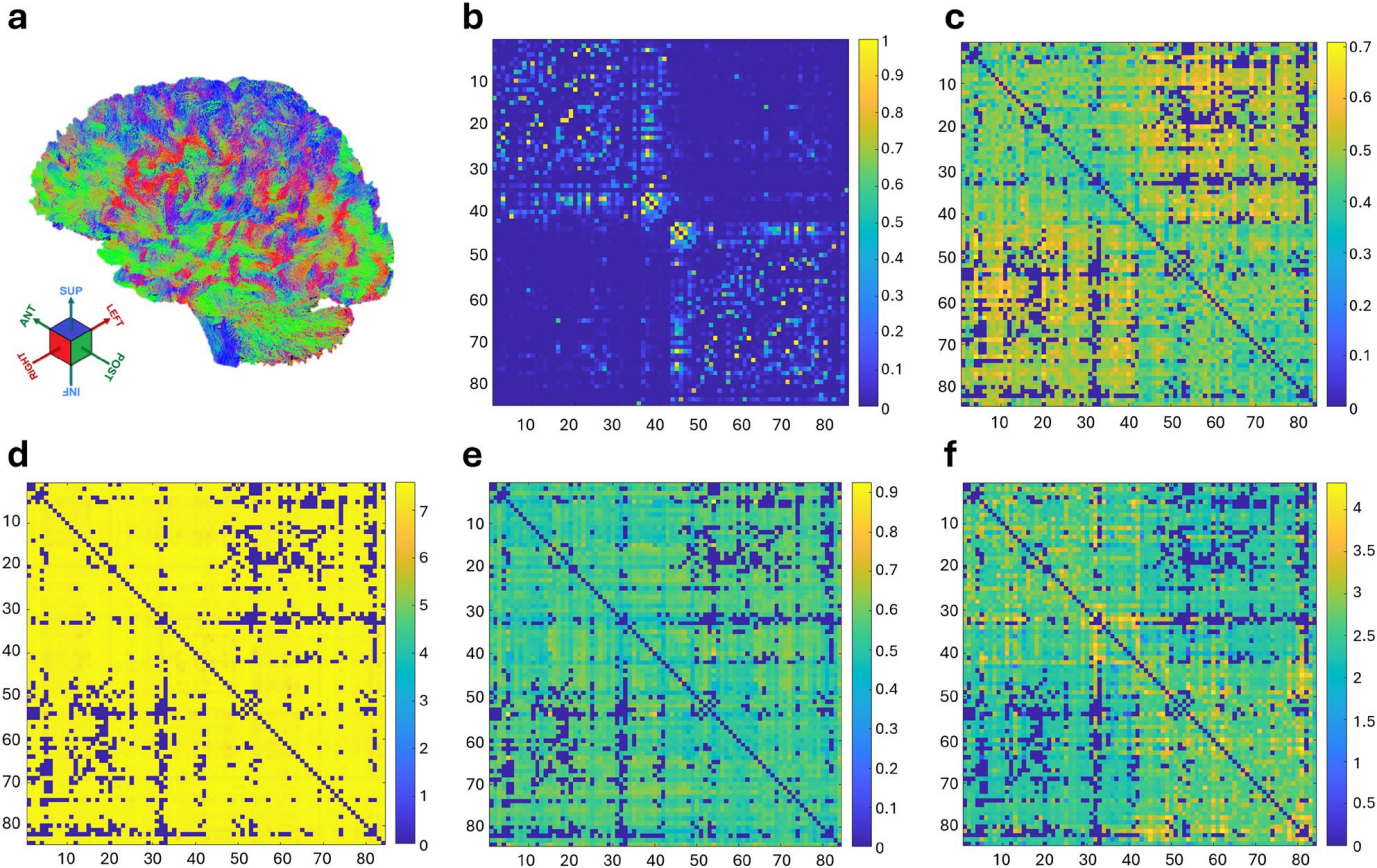
Tractogram and microstructure-weighted connectomes matrices from a healthy subject. Each matrix has eighty-four entries, corresponding to distinct brain regions as defined by the FreeSurfer parcellation based on the Desikan-Killiany atlas^57^. Regions 1-42 correspond to the left hemisphere, while regions 43-84 correspond to the right hemisphere. Matrix elements represent the reconstructed structural connections between regions, modulated with a specific parameter. The negative logarithmic transformation was applied to MD- and ECVF- weighted connectomes. (**a**) Tractogram (**b**) NOS-weighted connectome (**c**) FA-weighted connectome (**d**) MD-weighted connectome (**e**) INVF-weighted connectome (**f**) ECVF-weighted connectome.

Four-shell protocol data were used to reconstruct microstructure-weighted connectomes in each subject, following the procedure explained in the Methods section. These connectomes were modelled as undirected weighted networks, using tensor and Bingham-NODDI parameters. Specifically, connectome edges were weighted with FA and MD from the tensor model, and INVF and ECVF from the Bingham-NODDI model. The ODI and $\beta$-fraction were excluded, as the reproducibility analysis showed high instability for these parameters. As a result, four distinct parameter-weighted connectomes were generated for each subject at each acquisition site. Additionally, NOS-weighted connectomes were extracted as reference. Fig. 6 shows an example of the tractogram and the connectomes extracted for one subject. In the parameter-weighted connectomes (c-f), matrix elements represent the reconstructed structural connections between regions modulated with a specific parameter. Considering the FA-weighted connectome, for example, the high matrix values in the inter-hemispheric quadrants represent regions connected with highly coherent WM tracts (i.e. with large median FA, such as the corpus callosum).

**Table 1:** Network metrics extracted from FA- and MD-weighted connectomes for each healthy volunteer (HV) in both acquisition sites. The coefficient of variation was computed separately between each pair of values and is reported in bold. The Bland-Altman bias and intraclass correlation coefficient (ICC) were computed for each metric across all volunteers.

		Density		Efficiency		Modularity		Clust. Coeff.		Mean Strength	
		Site 1	Site 2	Site 1	Site 2	Site 1	Site 2	Site 1	Site 2	Site 1	Site 2
FA	HV 1	0.859	0.867	0.447	0.461	0.032	0.031	0.593	0.601	33.835	35.096
		**0.47%**		**1.52%**		**3.27%**		**0.65%**		**1.83%**	
	HV 2	0.771	0.785	0.449	0.461	0.061	0.051	0.551	0.538	31.658	32.908
		**0.96%**		**1.30%**		**8.27%**		**1.19%**		**1.94%**	
	HV 3	0.748	0.784	0.423	0.432	0.080	0.070	0.545	0.568	29.273	30.788
		**2.36%**		**1.04%**		**7.10%**		**2.15%**		**2.52%**	
	HV 4	0.795	0.822	0.450	0.467	0.045	0.043	0.556	0.571	32.481	34.448
		**1.69%**		**1.94%**		**2.63%**		**1.24%**		**2.94%**	
	*BA bias*	-0.022		-0.013		0.006		-0.008		-1.498	
	*LoA*	(-0.046, 0.003)		(-0.020, -0.006)		(-0.003, 0.015)		(-0.039, 0.022)		(-2.156, -0.840)	
	*ICC*	0.86		0.69		0.93		0.78		0.76	
MD	HV 1	0.859	0.867	6.932	6.977	0.042	0.039	0.875	0.883	531.167	537.376
		**0.47%**		**0.33%**		**3.75%**		**0.42%**		**0.58%**	
	HV 2	0.771	0.785	6.628	6.700	0.063	0.068	0.829	0.836	478.243	488.669
		**0.96%**		**0.54%**		**3.62%**		**0.43%**		**1.08%**	
	HV 3	0.748	0.784	6.537	6.678	0.103	0.086	0.807	0.830	463.579	486.467
		**2.36%**		**1.06%**		**8.91%**		**1.44%**		**2.41%**	
	HV 4	0.795	0.822	6.729	6.839	0.062	0.045	0.853	0.864	494.266	511.822
		**1.69%**		**0.81%**		**15.42%**		**0.68%**		**1.74%**	
	*BA bias*	-0.022		-0.092		0.008		-0.012		-14.270	
	*LoA*	(-0.046, 0.003)		(-0.173, -0.010)		(-0.013, 0.029)		(-0.028, 0.003)		(-28.798, 0.259)	
	*ICC*	0.86		0.83		0.88		0.88		0.85

**Table 2:** Network metrics extracted from INVF- and ECVF-weighted connectomes for each healthy volunteer (HV) in both acquisition sites. The coefficient of variation was computed separately between each pair of values and is reported in bold. The Bland-Altman bias and intraclass correlation coefficient (ICC) were computed for each metric across all volunteers.

		Density		Efficiency		Modularity		Clust. Coeff.		Mean Strength	
		Site 1	Site 2	Site 1	Site 2	Site 1	Site 2	Site 1	Site 2	Site 1	Site 2
INVF	HV 1	0.859	0.867	0.512	0.525	0.030	0.029	0.525	0.596	38.923	40.144
		**0.47%**		**1.22%**		**2.46%**		**6.33%**		**1.54%**	
	HV 2	0.771	0.785	0.517	0.523	0.056	0.055	0.606	0.604	36.925	37.766
		**0.96%**		**0.56%**		**0.45%**		**0.22%**		**1.13%**	
	HV 3	0.748	0.784	0.496	0.505	0.092	0.075	0.529	0.633	34.674	36.410
		**2.36%**		**0.95%**		**10.42%**		**8.98%**		**2.44%**	
	HV 4	0.795	0.822	0.517	0.526	0.052	0.038	0.653	0.629	37.641	39.013
		**1.69%**		**0.85%**		**15.83%**		**1.91%**		**1.79%**	
	*BA bias*	-0.022		-0.009		0.008		-0.037		-1.292	
	*LoA*	(-0.046, 0.003)		(-0.015, -0.004)		(-0.009, 0.026)		(-0.156, 0.082)		(-2.018, -0.567)	
	*ICC*	0.86		0.68		0.89		0.12		0.76	
ECVF	HV 1	0.859	0.867	2.460	2.622	0.070	0.069	0.532	0.540	184.885	198.415
		**0.47%**		**3.20%**		**1.10%**		**0.78%**		**3.53%**	
	HV 2	0.771	0.785	2.334	2.620	0.094	0.094	0.463	0.523	162.356	185.485
		**0.96%**		**5.77%**		**0.24%**		**6.10%**		**6.65%**	
	HV 3	0.748	0.784	2.306	2.408	0.134	0.117	0.474	0.491	158.943	171.443
		**2.36%**		**2.16%**		**7.00%**		**1.73%**		**3.78%**	
	HV 4	0.795	0.822	2.354	2.663	0.082	0.062	0.515	0.545	168.561	195.199
		**1.69%**		**6.16%**		**13.63%**		**2.84%**		**7.32%**	
	*BA bias*	-0.022		-0.215		0.010		-0.029		-18.949	
	*LoA*	(-0.046, 0.003)		(-0.409, -0.021)		(-0.010, 0.030)		(-0.073, 0.016)		(-32.695, -5.203)	
	*ICC*	0.86		0.13		0.88		0.49		0.37	

Various network metrics were extracted from each weighted connectome, namely density, global efficiency, modularity, clustering coefficient and nodal strength. Tables 1 and 2 report the metrics obtained for each subject from tensor- and NODDI-weighted connectomes, respectively. All weighted connectomes exhibited identical density values for each subject, as this metric is, by definition, independent of edge weights. In contrast, the other network metrics exhibited different value ranges across the weighted connectomes, correctly reflecting their sensitivity to the applied weighting. Statistical analysis was performed to assess the inter-site reproducibility of network metrics extracted from parameter-weighted connectomes. The resulting CVs, Bland-Altman biases and ICCs are reported in Tab. 1 and Tab. 2. In FA-weighted connectomes, inter-site CVs for density, efficiency, clustering coefficient, and mean strength were substantially below the 5% threshold, with all values less than 3%. Conversely, modularity exhibited larger CVs, with peaks of 7-8% in two subjects. Minimal Bland-Altman biases were found for all the network metrics, including modularity. Finally, the ICCs demonstrated excellent reliability^69^ for modularity, good reliability for density, clustering coefficient, and mean strength, and moderate reliability for efficiency. MD-weighted connectomes showed small BA biases and low inter-site CVs across metrics, with the exception of modularity, which exceeded the threshold in two volunteers (CV equal to 8.91% for HV3 and 15.42% for HV4). Good reliability was observed for all the metrics based on the computed ICCs. INVF-weighted connectomes displayed similar patterns for all the metrics except for the clustering coefficient, which exhibited CVs above the 5% threshold in two volunteers (HV1 and HV3) and poor reliability (ICC = 0.12). Finally, network metrics derived from ECVF-weighted connectomes showed poor reproducibility. Modularity exhibited a broad range of CVs across subjects, spanning from 0.24% (HV2) to 13.63% (HV4). Furthermore, efficiency, clustering coefficient, and mean strength displayed some CVs above the 5% threshold, along with low ICC values ($<0.5$) indicating poor reliability. Bland-Altman analysis for these metrics demonstrated low variability, excluding the presence of a bias between acquisition sites.

## Discussion

Microstructure-weighted connectomes provide a framework for exploring human brain organisation and function. Graph metrics extracted from these connectomes have shown sensitivity to pathological changes in multiple neurological disorders^19–27^ and exhibit significant associations with functional connectivity^18^. The reconstruction of microstructure-weighted connectomes involves a long and complex processing pipeline, which may introduce variability and potentially affect the reliability of the derived network metrics^1,13,28,29^. Therefore, assessing the reproducibility of these connectomes is essential to establish their validity and ensure their suitability for both research and clinical purposes. While several studies have evaluated the robustness of NOS-based connectomes and the derived network metrics^28,30–33^, to our knowledge, this is the first study which comprehensively investigates the reproducibility of connectomes weighted with parameters derived from tensor and Bingham-NODDI models. Specifically, we provide the first assessment of the robustness of the entire microstructure-weighted connectome reconstruction pipeline, verifying temporal, inter-site and inter-protocol consistency of diffusion model parameters used as weights and inter-site stability of the extracted network metrics. A four-shell HARDI acquisition scheme was tested and employed to reconstruct microstructure-weighted connectomes, as it provides more accurate fibre modelling compared to two-shell schemes^38^.

Overall, results extracted from four-shell protocol data demonstrated good reproducibility. DTI parameters exhibited low temporal variability in the phantom ring and crossing ROIs, with most CVs equal to 0% when considering two significant digits. This aligns with previous findings from single-shell protocols^71,72^ and further extends these results to multi-shell protocols, including those with b-values greater than 1000 s/mm$\vphantom{0}^2$. Regarding the parameter values, FA was found to be $0.70 \pm 0.08$ in the rings region, in accordance with what has been declared by the phantom manufacturer ($0.78 \pm 0.02$)^41^. In the crossing region of the two fibre strands, instead, FA remained stable around 0.5, not reflecting the true phantom characteristics, in which FA does not decrease. On the contrary, in these regions FA is artificially low as it reflects and confirms the inability of the DTI model to take into account crossing fibres^73,74^, even with multi-shell data. High temporal stability was also observed for ICVF and INVF extracted from the Bingham-NODDI fit on four-shell protocol data in both phantom ROIs. These findings align with previous *in vivo* studies employing two-shell^39^ and three-shell protocols^75^. The ICVF was found to be close to 1 in all the scans, correctly reflecting the presence of fibres and the absence of free water in the ROIs considered. The ODI was found to be close to 0 in both the ROIs, indicating the high compactness and strong directionality of the phantom fibres. Concerning temporal stability, ODI exhibited greater variability in the crossing ROI compared to other parameters (CV = 4.19%). Furthermore, its CV exceeded the 5% threshold when fitting two-shell data in the same region (CV = 7.07%). This greater variability may stem from the design of the Bingham-NODDI model, which only accounts for a single fibre population and therefore cannot accurately interpret crossing fibres^37,76^. The larger MSE found in the crossing region compared to the ring region further supports this hypothesis. Finally, the $\beta$ concentration parameter showed the poorest temporal stability (CV = 9.18% in the ring ROI) and large standard deviations, suggesting the use of the other metrics when performing analyses with the NODDI model. Four-shell protocol results were then investigated for inter-site consistency. Analysis of DTI parameters revealed good stability and non-systematic differences among sites across all the brain ROIs. These findings further confirm the reliability of the DTI parameters, as found in literature for single-shell^77^. INVF and ICVF parameters from Bingham-NODDI exhibited low intra-subject variability, with CVs below 2.5% across all brain ROIs, supporting their use as weights for connectome edges. Conversely, although $\beta$ concentration parameter and ODI generally exhibited small inter-site fluctuations, there were a few instances in which the CV exceeded the 5% threshold. As a consequence, their intra-subject reproducibility appears less robust compared to that of the other parameters. Based on these observations and prior findings from the temporal stability study, ODI and $\beta$ parameters were excluded from the subsequent microstructure-weighted connectome reconstruction. This was done in order to prevent variations in connectome network metrics caused by fluctuations of the parameters used to weight the edges. In summary, our findings identify FA and MD from the DTI model, alongside INVF and ICVF from the Bingham-NODDI model, as the most reproducible parameters within the investigated framework. These metrics quantify the microstructural properties and integrity of fibres and surrounding tissues, thereby providing a more biologically meaningful weighting for connectomes compared to NOS-based weights^13^.

The choice of the diffusion acquisition protocol can affect differently the acquired SNR and imaging artifacts^75^, which, in turn, can influence the parameters extracted from diffusion models^78^. Protocols with two distinct b-values are recommended for fitting the NODDI model^37^. As a four-shell protocol was used in this study, it was important to assess the accordance of the obtained results with those extracted from the conventional two-shell protocol. Additionally, protocol comparison also included tensor model parameters. The comparison was performed with both phantom and *in vivo* data to investigate the accordance under ideal and real-world conditions. Four-shell and two-shell protocols generally provided comparable results. Statistical analysis of tensor model parameters demonstrated good agreement between the protocols in the two phantom ROIs and *in vivo* in the corpus callosum and thalamus. In contrast, worse accordance was found for both FA and MD in the ventricles ROI, with CVs of 5-6%. Therefore, although these parameters showed low within-protocol variability in this ROI, greater caution is needed when comparing values across different protocols. Notably, FA values in the ventricles ROI did not exhibit an observable Bland-Altman bias, with the 95% CI including zero. Conversely, a positive bias was found for MD, suggesting a systematic larger estimation of the diffusivity when using the two-shell protocol. Concerning Bingham-NODDI parameters, INVF and ICVF extracted from phantom data were highly consistent across protocols in both ROIs. Larger inter-protocol fluctuations were observed for $\beta$, possibly due to its previously reported poor temporal stability within protocols and its large standard deviation. Similarly, the protocol disagreement observed for the ODI in the crossing region may stem from its limited reproducibility previously found in the same ROI. As mentioned earlier, these inconsistencies may reflect the limitations of Bingham-NODDI in modelling regions with crossing fibres, highlighting the need for more advanced formulations that account for the complexities introduced in these regions. Four-shell and two-shell protocols showed strong agreement and minimal systematic bias on *in vivo* data for all NODDI parameters in both WM and GM ROIs. However, the high inter-protocol variability observed in the phantom suggests caution when comparing $\beta$ and ODI parameters across studies using different multi-shell protocols. Notably, INVF values demonstrated good consistency, in contrast to previous studies that reported variability when changing the outer-shell b-value^78^. These findings confirm that the Bingham-NODDI model does not benefit from the inclusion of additional shells, as previously reported^37^.

Microstructure-weighted connectomes were constructed using data acquired with the four-shell protocol. Based on the reproducibility analysis presented above, only FA, MD, INVF and ECVF (the complement of ICVF) were used to weight connectome edges. As a result, four distinct types of microstructure-weighted connectomes were generated for each subject at each acquisition site. Various network metrics were extracted from each type of weighted connectome, measuring network integration and segregation. Overall, these metrics proved to be sensitive to the weighting parameter and correctly captured inter-subject variability. The results were generally consistent with those reported in previous studies for healthy subjects^22^, despite the use of a different orientation distribution function for the NODDI model. The reliability of network metrics extracted from microstructure-weighted connectomes was evaluated by assessing their inter-site reproducibility. Overall, density, efficiency, clustering coefficient and mean strength derived from FA- and MD-weighted connectomes showed high reproducibility, in accordance with previous findings for FA^35^. In contrast, modularity exhibited a more complex behaviour. While it demonstrated good reliability (ICC = 0.93 and ICC = 0.88 for FA and MD, respectively) and minimal BA biases, its inter-site CVs frequently exceeded the 5% threshold. This suggests that, although intra-subject variability remains lower than inter-subject variability (as given by the ICCs), the absolute intra-subject variability (captured by the CVs) is non-negligible. Likewise, modularity presented large intra-subject fluctuations in INVF-weighted connectomes. These consistent findings suggest that modularity may be particularly sensitive to minor variations in edge weights or to variability introduced during the complex connectome construction process. Therefore, our findings suggest that modularity derived from FA-, MD- and INVF-weighted connectomes should be used with caution in future studies. Notably, previous work has reported good reproducibility for modularity when derived from NOS-based connectomes^28^. Unlike FA and MD, INVF-weighted connectomes exhibited poor reliability also for the clustering coefficient, mostly driven by higher single-subject variability. Finally, network metrics derived from ECVF-weighted connectomes generally showed larger instability compared to those extracted from other weightings, possibly suggesting that the other parameters may be more suitable for connectome weighting in future applications. To summarise, our analysis identifies FA-, MD- and INVF-weighted connectomes as the most stable frameworks, with density, global efficiency, clustering coefficient and mean strength emerging as the most reliable metrics for investigating brain network topology.

Taken together, this study highlights the reliability of the microstructure-weighted connectome reconstruction pipeline, identifying the most robust weighting strategies. Unlike standard NOS-based approaches, these connectomes integrate biological information derived from diffusion parameters, offering a richer characterisation of brain connectivity^13^. For instance, the inclusion of Bingham-NODDI parameters enriches the connectome with specific information on tissue compartments and axonal integrity. Consequently, microstructure-weighted connectomes hold the potential for higher sensitivity and specificity to pathological alterations in neurological diseases such as multiple sclerosis^19–23^, Alzheimer’s disease^26^, Parkinson’s disease^24^ and schizophrenia^27^. Different weighting strategies provide unique descriptions of brain structure and connectivity^79^. Thus, network metrics derived from these weighted connectomes can be combined to characterise disease patterns, ultimately paving the way for their use as clinical biomarkers.

While promising, these findings should be considered preliminary due to the limited sample size, which precludes a robust assessment of confounding effects associated with inter-subject variability. Therefore, validation in larger cohorts is required to allow for formal hypothesis testing and confirm these results with greater statistical power. Specifically, a sample size of n $\ge$ 30 is recommended^69^ to ensure robust reliability estimates, although this represents a challenging target for this type of study. Additionally, future studies should extend this analysis to multi-vendor datasets, using harmonisation methods (e.g. ComBat^80^) to mitigate scanner effects. Furthermore, given the known impact of parcellation resolution on network metrics^81^, the reproducibility of microstructure-weighted connectomes should also be verified across different nodal scales. Finally, our phantom analysis confirmed the limitations of DTI and Bingham-NODDI in modelling crossing fibre configurations. Consequently, future work should consider adopting alternative advanced models, such as the spherical mean technique (SMT)^76^, to estimate microstructural parameters.

In conclusion, this study represents a significant step toward the validation of microstructure-weighted connectomes, demonstrating their feasibility and supporting their broader application. By propagating diffusion model parameters through the network, microstructure-weighted connectomes offer a richer characterisation of structural connectivity. Different weighting strategies may highlight distinct aspects of brain organisation, allowing graph metrics to capture specific patterns of network alterations in neurological disorders.

## Supplementary Information

Supplementary Information.

## Data Availability

Data are available from the corresponding author upon reasonable request.
