# Supplementary material for: A pilot study on protocol consistency and graph metric reproducibility in microstructure-weighted connectomes
Source: Sci Rep. 2026 Feb 11;16:8288. doi: 10.1038/s41598-026-38964-z (PMC12966391; doi:10.1038/s41598-026-38964-z)
Supplement: Supplementary file 1 — Supplementary Information. [file 41598_2026_38964_MOESM1_ESM.pdf]

A pilot study on protocol consistency and graph metric reproducibility in microstructure-weighted connectomes

Maddalena Cavallo[1, 2], Mattia Ricchi[2, 3, 4], Aaron Axford[2], Kylie Yeung[2, 5, 6], Jordan McGing[2], Leonardo Brizi[1, 4], Damian J. Tyler[2, 7], Claudia Testa[1, 4, +], and James T. Grist[2, 6, +, *]

[1]Department of Physics and Astronomy, University of Bologna, Bologna, Italy
[2]Oxford Centre for Clinical Magnetic Resonance Research, University of Oxford, Oxford, UK
[3]Department of Computer Science, University of Pisa, Pisa, Italy
[4]National Institute of Nuclear Physics (INFN), Division of Bologna, Bologna, Italy
[5]Department of Oncology, University of Oxford, Oxford, UK
[6]Department of Radiology, Oxford University Hospitals NHS Trust, Oxford, UK
[7]Department of Physiology, Anatomy, and Genetics, University of Oxford, Oxford, UK
[*]james.grist@cardiov.ox.ac.uk
[+]these authors contributed equally to this work

SUPPLEMENTARY MATERIAL

Intraclass correlation coefficients

	FA	MD	INVF	ICVF	β	ODI
Corpus callosum	0.90	0.62	–	–	–	–
Genu C.C.	–	–	0.96	0.79	0.96	0.79
Splenium C.C.	–	–	0.95	-0.05	0.91	0.86
Ant. limb I.C.	–	–	0.80	0.02	0.97	1.00
Post. limb I.C.	–	–	0.70	0.12	0.92	0.89
Thalamus	0.64	0.65	0.35	0.86	0.82	0.96
Caudate	–	–	0.49	0.97	0.35	0.79
Putamen	–	–	0.44	0.20	0.90	0.85
Ventricles	0.88	0.96	–	–	–	–

Table S1. Inter-site intraclass correlation coefficients of DTI and NODDI parameters in *in vivo* ROIs. FA: fractional anisotropy, MD: mean diffusivity, INVF: intra-neurite volume fraction, ICVF: intra-cellular volume fraction, β: concentration parameter, ODI: orientation dispersion index, C.C.: corpus callosum, I.C.: internal capsule.

Bland-Altman analysis

		Genu C.C.	Splenium C.C.	Ant. limb I.C.	Post. limb I.C.	Thalamus	Caudate	Putamen
INVF	BA bias	-0.0035	-0.0041	0.0007	-0.0116	0.0075	0.0079	0.0040
	Lower LoA	-0.0255	-0.0277	-0.0130	-0.0142	0.0018	-0.0061	-0.0133
	Upper LoA	0.0185	0.0194	0.0144	-0.0090	0.0132	0.0218	0.0212
ICVF	BA bias	0.0085	0.0062	-0.0026	0.0089	-0.0011	0.0049	0.0003
	Lower LoA	-0.0183	-0.0006	-0.0164	-0.0064	-0.0039	-0.0142	-0.0011
	Upper LoA	0.0353	0.0129	0.0111	0.0241	0.0017	0.0241	0.0017
β	BA bias	-0.0137	0.0032	0.0247	0.0322	0.0013	0.0173	0.0254
	Lower LoA	-0.0539	-0.0303	-0.0033	-0.0124	-0.0179	-0.0251	0.0051
	Upper LoA	0.0265	0.0367	0.0526	0.0768	0.0205	0.0596	0.0457
ODI	BA bias	-0.0012	0.0002	-0.0055	-0.0061	-0.0027	-0.0102	-0.0051
	Lower LoA	-0.0091	-0.0024	-0.0115	-0.0179	-0.0057	-0.0194	-0.0131
	Upper LoA	0.0068	0.0027	0.0006	0.0056	0.0002	-0.0010	0.0028

Table S2. Bland-Altman analysis of Bingham-NODDI parameters for multi-shell protocol comparison from Site 1. INVF: intra-neurite volume fraction, ICVF: intra-cellular volume fraction, β: concentration parameter, ODI: orientation dispersion index, C.C.: corpus callosum, I.C.: internal capsule, LoA: limits of agreement.

		Genu C.C.	Splenium C.C.	Ant. limb I.C.	Post. limb I.C.	Thalamus	Caudate	Putamen
INVF	BA bias	0.0079	-0.0001	0.0016	-0.0114	0.0077	0.0075	0.0080
	Lower LoA	-0.0011	-0.0114	-0.0036	-0.0240	0.0032	0.0026	0.0055
	Upper LoA	0.0169	0.0113	0.0069	0.0012	0.0121	0.0124	0.0105
ICVF	BA bias	-0.0014	0.0075	-0.0011	0.0075	0.0010	0.0013	0.00004
	Lower LoA	-0.0162	-0.0079	-0.0064	-0.0056	-0.0049	-0.0080	-0.00016
	Upper LoA	0.0135	0.0229	0.0041	0.0205	0.0069	0.0106	0.00024
β	BA bias	0.0015	-0.0069	0.0217	0.0322	0.0027	0.0097	0.0187
	Lower LoA	-0.0309	-0.0298	-0.0087	0.0077	-0.0122	-0.0686	-0.0108
	Upper LoA	0.0340	0.0161	0.0521	0.0567	0.0177	0.0880	0.0483
ODI	BA bias	-0.0042	-0.0028	-0.0039	-0.0071	-0.0032	-0.0111	-0.0022
	Lower LoA	-0.0071	-0.0062	-0.0093	-0.0137	-0.0081	-0.0243	-0.0251
	Upper LoA	-0.0013	0.0007	0.0016	-0.0005	0.0017	0.0022	0.0207

Table S3. Bland-Altman analysis of Bingham-NODDI parameters for multi-shell protocol comparison from Site 2. INVF: intra-neurite volume fraction, ICVF: intra-cellular volume fraction, β: concentration parameter, ODI: orientation dispersion index, C.C.: corpus callosum, I.C.: internal capsule, LoA: limits of agreement.
